# Single-Component Starch-Based Hydrogels for Therapeutic Delivery

**DOI:** 10.3390/molecules29225463

**Published:** 2024-11-20

**Authors:** Alfio Pulvirenti, Antonella Caterina Boccia, Carolina Constantin, Mihaela Surcel, Adriana Munteanu, Victor-Eduard Peteu, Monica Neagu

**Affiliations:** 1 Istituto di Scienze e Tecnologie Chimiche (SCITEC) “Giulio Natta”, C.N.R., Via Alfonso Corti 12, 20133 Milano, Italy; alfio.pulvirenti@scitec.cnr.it; 2 “Victor Babes” National Institute of Pathology, 99-101 Splaiul Independenței, 050096 Bucharest, Romania; caroconstantin@gmail.com (C.C.); msurcel2002@yahoo.com (M.S.); iadinuta@yahoo.com (A.M.); peteuvictoreduard@gmail.com (V.-E.P.); neagu.monica@gmail.com (M.N.); 3 Colentina Clinical Hospital, 19-21, Sos Stefan Cel Mare, 020125 Bucharest, Romania; 4 Doctoral School, Politechnica University of Bucharest, 313 Splaiul Independenței, 060042 Bucharest, Romania

**Keywords:** hydrogels, starch, NMR, flow cytometry, cytotoxicity, therapeutic delivery

## Abstract

Hydrogels are interesting materials as delivery systems of various therapeutic agents, mainly due to the water-swollen network and the localized and sustained drug release. Herein, single-component starch-based hydrogels with enhanced degradation rates were produced by applying a facile synthesis and proposed for a novel delivery system of therapeutic molecules. Starch was oxidized with sodium periodate in water and mild conditions to generate aldehyde derivatives that, after a freeze-thaw procedure, were allowed to compact and stable hydrogels. Oxidized starch was also cross-linked with asparagine through a Schiff base reaction to link the active molecule directly to the polysaccharide structure. The materials were structurally and morphologically characterized, and the ability to adsorb and release over time an active molecule was proven by qNMR spectroscopy. The cytotoxicity was evaluated on CAL-27 cell line (oral squamous cell carcinoma). Results indicated that synthesized hydrogels lead to a “frozen proliferative” state on cells due to the swelling capability in the cell medium. This behavior was confirmed by flow cytometry data indicating the hydrogels induced less “early apoptosis” and more “late apoptosis” in the cells, compared to the untreated control. Since the proposed materials are able to control the cell proliferation, they could open a new scenario within the field of precise therapeutic applications.

## 1. Introduction

The great progress of hydrogel systems for therapeutic applications is significantly transforming the landscape of medicine due to their versatile, highly tunable properties and breakthroughs in biomaterial technologies [1,2,3,4].

Hydrogels are three-dimensional cross-linked materials characterized by a porous structure capable of retaining a huge amount of water or biological fluids [5]. Refining the structural and compositional aspects, responsive hydrogels with enhanced biocompatibility and biodegradability can be designed to develop ideal platforms for therapeutic delivery and encapsulation of cells, drugs or composite drugs, and active agents that can be tailored with high precision for desired applications [5,6,7,8,9,10].

Over the years, hydrogels have been explored for various therapeutical applications, such as targeted therapy aiming to promote tissue regeneration [11,12]; to enable the fabrication of complex, biomimetic tissue or scaffold [13,14], as well as innovative solutions for wound healing and inflammatory disease treatment [15,16,17,18]. The therapeutic hydrogels developed to date possess the unprecedented ability to localize the delivery and release of drugs to the target site of interest, reducing the drug’s exposure and minimizing systemic side effects. To diversify and enlarge the performance of the early generation of hydrogels, multicomponent materials have been designed and used to synthesize hydrogels and to selectively tailor the material properties and overcome the disadvantages limiting the delivery of poor water-soluble molecules, thus opening their use to new therapeutic and attractive approaches. Typically, the multicomponent hydrogels have been produced from synthetic polymers, such as PEG-PLA copolymers (poly(ethylene glycol)-poly(lactide)) [19,20], PLGA (poly(lactic-co-glycolic acid)) [21,22], PEG−PCL (poly(ethylene glycol) poly(ε-caprolactone)) [23,24], PEO (poly(ethylene oxide)), PPO-PEO (poly(propylene oxide)-poly(ethylene oxide)) [25,26], poly (2-hydroxyethyl methacrylate) (PHEMA) [27], poly (acrylic acid) (PAA) [28,29], and poly (acrylamide) (PAAm) polyurethane [30,31].

The growing interest in finding more biocompatible solutions has also pushed toward the research of multicomponent hydrogels based on proteins and peptides, thus promoting the development of materials able to respond to external signals, such as variations in small molecules, pH and temperature, or light [32]. Multicomponent hydrogels based on protein, such as collagen [33], gelatin [34], protein-engineered triblock copolymers [35], elastin-based polypeptides [36,37,38], and other protein self-assembled hydrogels, were produced for regenerative therapy applications [39,40,41,42].

Polysaccharides, as natural polymers, largely available, are intrinsically biocompatible, biodegradable, and nontoxic, and for these raisons have been applied for producing hydrogels. Polysaccharide-based hydrogels have been mainly developed from sodium hyaluronate, chitosan, cellulose, polygalactomannans, alginate, dextran, pullulan, hyaluronic acid, pectin, cellulose, hemicellulose, chitosan, k-carrageenan and starch, and some of them as, the new generation of hydrogel-based on polysaccharides, has received the approval of European Medicines Agency (EMA) and/or Food and Drug Administration (FDA) to be tested in the clinical practise [43,44,45,46,47,48,49,50,51,52].

Starch is the second largest material available from biomass among the polysaccharides and has been used as the key formulation ingredient for manufacturing therapeutical applications dedicated to wound repair, the delivery of growth factors [6], cell stems [6], bioactive agents [7], drugs [6,8], and antimicrobial, anti-inflammatory and antioxidant agents [53]. Starch is not the most popular polysaccharide used to produce single-component hydrogels for therapeutical treatment, as the materials suffer several restrictions due to the fast degradation rate coupled with low mechanical properties, resulting in hydrogels characterized by a weak structure and limited water-holding attitude [54,55]. To circumvent these limitations, several strategies have been explored for the production of starch-based hydrogels, which consist of mixing or blending starch with polymers or crosslinking agents to produce multinetwork hydrogels, chemically modifying starch, or using ionic radiation [56,57,58,59]. Considering that the use of chemicals in the final materials may limit the applications of hydrogels due to health and environmental concerns, the challenge of developing sustainable manufacturing techniques for producing functionalized starch-based hydrogels needs to be addressed [54].

Therefore, we have recently developed single-component hydrogels, modifying starch from pea pods and potato peels, by applying a green synthetic approach and a freezing-thawing procedure, as well as asparagine crosslinked starch hydrogels applying a Schiff-base reaction. The newly developed formulations, intended as single components, were manufactured according to a simple, economic, and environmentally friendly method, achievable by a large-scale process and without using chemicals limiting the hydrogel applications. The obtained materials showed good mechanical stability and strength and good water retention capability. Furthermore, the synthesized hydrogels were found to be intrinsically capable of inhibiting the growth of some gram-positive and gram-negative bacterial strains [60].

Inspired by these findings, in this work, the single-component starch-based hydrogels were incorporated or crosslinked with bioactive molecules, and the ability to deliver them in such a sustained and controlled manner was proven by solution nuclear magnetic resonance spectroscopy (NMR). Tests on CAL-27 cell cultures (oral squamous cell carcinoma), such as lactate dehydrogenase (LDH) activity and metabolic activity (MTS) assays, were conducted to acquire preliminary information on the general toxicity of the device and to determine any biological damage caused by the contact of cells with the hydrogels, and successively, any effects produced by the cells were observed through flow cytometry technique. The novel formulation of hydrogels is expected to experience better adaptability to the site of administration, together with high biocompatibility, lowering the risks of infection and inflammation.

## 2. Results

### 2.1. Synthesis and Characterisation of Starch-Based Hydrogels

**Scheme 1:**
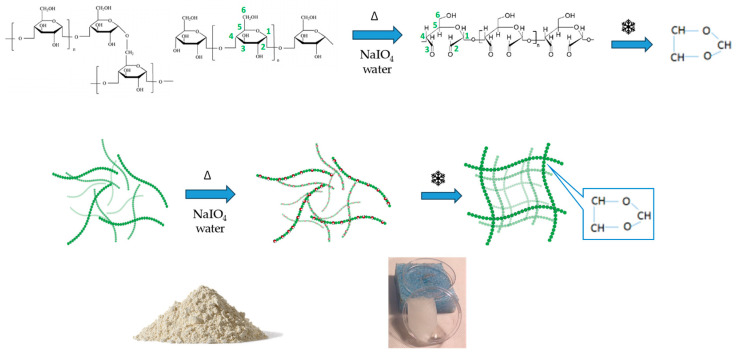
From native starch to hydrogel starch: the design of experiment.

Starch is a carbohydrate polymer characterized by the presence of two macromolecules, amylose and amylopectin, whose ratio may vary according to the source of origin [61]. Amylose is a linear polysaccharide composed entirely of D-glucose units joined by the α-1,4-glycosidic linkages and a relatively low molecular weight (~10^5^–10^6^ Da), while amylopectin is a branched-chain polysaccharide with glucose units linked primarily by α-1,4-glycosidic bonds that are connected through α-1,6-glycosidic bonds responsible for the branched structure. In this study, starch from two different plant wastes was used, pea pods and potato peels, respectively, for producing hydrogels after a chemical oxidation reaction. The chemical composition of starch, estimated by nuclear magnetic resonance spectroscopy (NMR), was found to be 67:33 for pea starch and 20:80 for potato starch [62]. Native starches were modified by oxidation with sodium periodate (NaIO_4_), able to selectively convert the C2 and C3 hydroxyl groups (-OH) on the pyranose ring into aldehyde groups (-CHO), as shown in Scheme 1.

**Figure 1:**
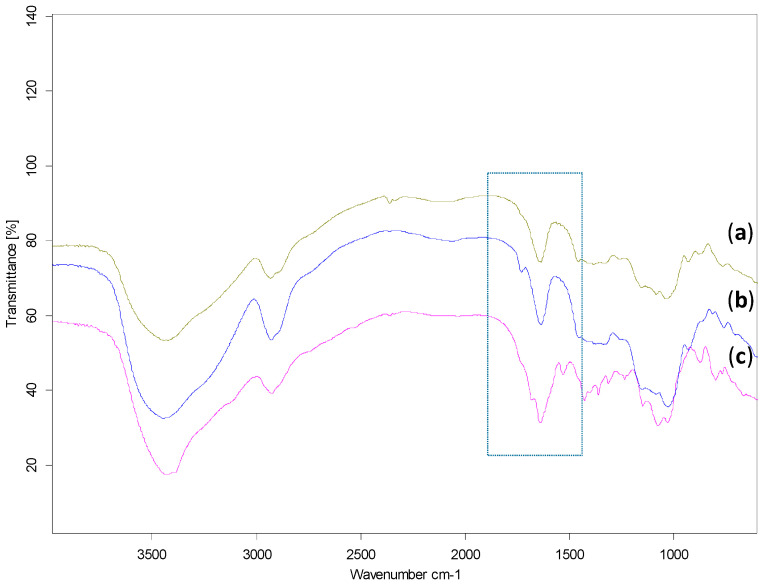
FTIR spectra of: (a) native pea starch; (b) dialdehyde starch; (c) crosslinked starch with asparagine. In the rectangle are the highlighted stretching vibrations of C=O and C=N, as newly formed groups.

The oxidized starches were structurally characterized by Fourier transform infrared spectroscopy (FTIR) to analyze the products deriving from the oxidation reaction. Data in Figure 1b confirmed the occurrence of the starch oxidation, due to the appearance of a new peak at 1730 cm^−1^ associated with the symmetric stretching vibration of the carbonyl group, not present in the FTIR spectra of native starch (Figure 1a and Figure S2).

**Figure 2:**
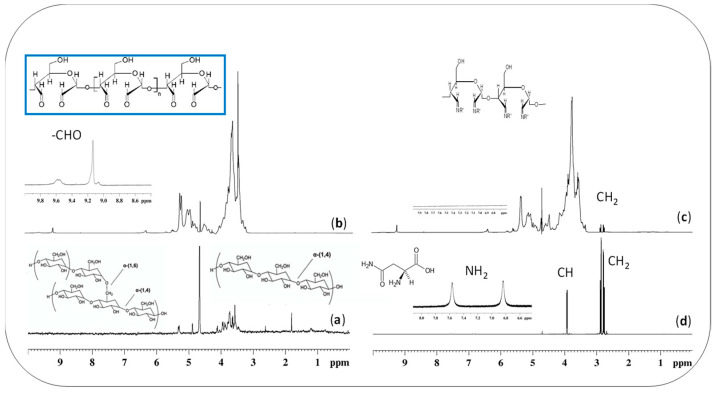
^1^H spectra of: (**a**) native starch; (**b**) oxidized starch; (**c**) crosslinked starch-asparagine; (**d**) free asparagine, @500 MHz in D_2_O.

The oxidized materials were also structurally characterized by solution NMR spectroscopy (in Figure 2), which confirmed the oxidation of starch into dialdehyde derivatives due to the presence of characteristic resonances at 9.8–9.1 ppm in the proton spectrum (Figure 2b).

Starch dialdehyde derivatives were then frozen at −18 °C to render a three-dimensional structure that, after a thawing step, resulted in stable and compact hydrogels, **AH** and **BH** (pea and potato starch-based hydrogels, respectively), as shown in Scheme 1 and illustrated in Figure S3.

**Scheme 2:**
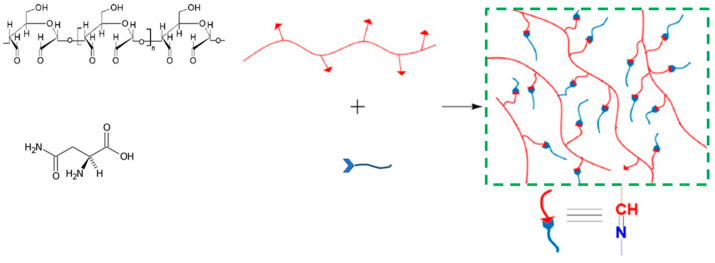
Asparagine crosslinked starch hydrogel.

As we were also interested in anchoring an active molecule directly to the polysaccharide matrixes, starch dialdehydes were modified with asparagine by applying a Schiff-base reaction that enabled the occurrence of a new covalent imine bond (R-C=N-), deriving from the reaction of the carbonyl group of dialdehyde (C=O) with the amino group of asparagine (-NH_2_). The success of the reaction was confirmed by FTIR spectroscopy by the appearance in Figure 1c of a characteristic imine band at 1680 cm^−1^, and by NMR spectroscopy (Figure 2c,d) following the disappearance of the asparagine protons on amine and the appearance of a new resonance associated to the formed imine group. After the crosslinking of asparagine to the starch dialdehyde, the derivatives were frozen at −18 °C and then thawed, as above reported for the nonderivatized hydrogels, to allow the obtainment of new starch-asparagine hydrogels (Scheme 2).

The idea to synthesize two groups of hydrogels, such as starch-based hydrogels **AH** and **BH**, and crosslinked starch-based hydrogels, **Basp**, was to screen the cells response after the contact with unloaded hydrogels, known to have antimicrobial activities against some gram-positive and negative bacterial strains, and to compare the results with those deriving from the cells contact with hydrogels having an active molecule, asparagine, directly bonded to the material [63].

**Figure 3:**
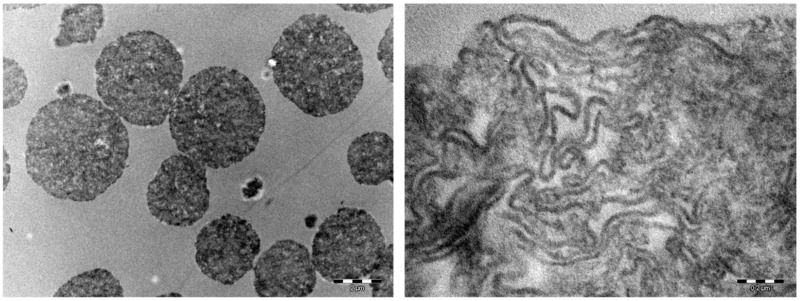
Transmission electron microscopic images of cryogel granules of various diameters, mean diameter 200 µm (on the **left**); cryogel structures consisting of long coiled starch fibers (on the **right**).

Hydrogels were microstructurally characterized by transmission electron microscopy analysis (TEM) (after lyophilization) to obtain real-space images of the hydrogels structure, considering that for these materials, multiple important properties in the biomedical domain depend on the structure resulting from bonding, orientation of fibers, and pores between the fibers [64]. Figure 3 shows granules of various diameters, whose mean diameter was determined to be approximately 200 µm. Analyzing in detail the images, it was possible to note that the intimate structure of synthesized materials consists of long coiled starch fibers with spaces that can harbor guest molecules to be delivered on site. This attitude of prepared hydrogels to act as carriers of active molecules was experimentally proved and illustrated in Section 2.3.

The pores average size has been evaluated by SEM analysis to be approximately 200 µm for sample **AH** and 250 µm for sample **BH**. Furthermore, the analysis indicated that both polymers exhibited a compact solid structure with nonhomogeneous pores and cavities [63].

### 2.2. Swelling Degree (SD)

**Figure 4:**
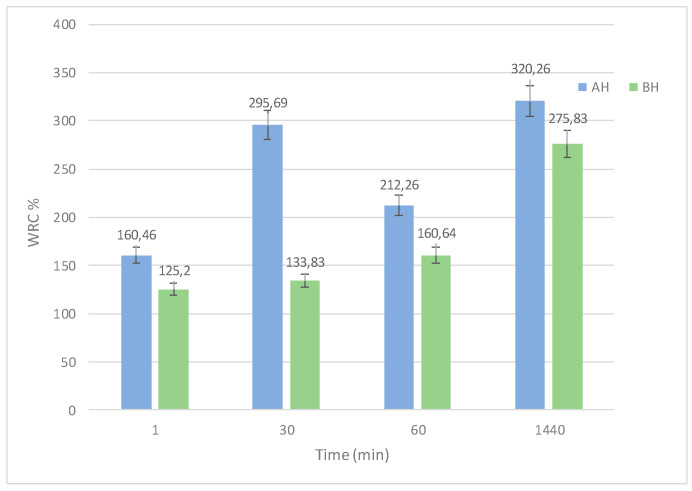
Swelling degree of samples **AH** and **BH**.

The swelling degree (SD) represents the capability of squeezed hydrogels to retain water and represents an important parameter to be considered for drug carrier applications. SD, after 60 s of immersion, was found to be higher for the **AH** sample than for the **BH** sample (results in Figure 4).

After 24 h, the SD value in Figure 4 has almost quadrupled for **AH** and slightly more than tripled for **BH,** indicating that there are no significant differences ascribed to the starch ratio characterizing **AH** and **BH** samples [65,66]. The swelling degree of the hydrogel crosslinked with asparagine was not evaluated over time, as it was found to be structurally fragile.

### 2.3. Cellular Behavior in the Presence of Hydrogels

To successfully design hydrogels for therapeutic delivery, it is crucial to consider the cell behaviors as a response to the hydrogel. The cell line CAL27, that is *per se* a keratinocyte, was chosen as a model cell line to cover any potential utilization also associated with desirable antiproliferative effects and was evaluated by conducting the LDH and MTS assays.

#### 2.3.1. LDH and MTS Assays

**Figure 5:**
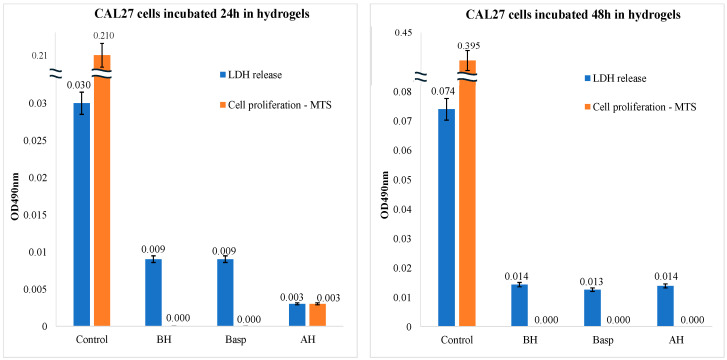
CAL-27 cell line in the presence of synthesized hydrogels. LDH and MTS assays, after 24 h of incubation (on the **left**) and after 48 h of incubation (on the **right**) in **AH**, **BH**, and **Basp** (pea, potato, and asparagine-crosslinked starch hydrogel) compared to control (untreated cells).

The cell viability assay, referring to the number of live, healthy cells in a sample, was carried out to investigate the effects of synthesized materials on the CAL-27 cell line. The LDH and MTS data in Figure 5 showed a decrease in cell proliferation accompanied by a blocking of the LDH release after 24 h incubation with the hydrogels **AH**, **BH**, and **Basp**. This behavior could be ascribed to the attitude of hydrogels to swell in the cell culture medium, generating a sort of gel able to protect the cell membrane against lysis and to limit, at the same time, the cell’s proliferation. Differences within **AH**, **BH,** and **Basp** can be ascribed to the greater stability (meaning the slower swellability) of pea starch hydrogels with respect to the potato and to the crosslinked hydrogels, which is a direct consequence of starch composition.

Furthermore, comparing the data in Figure 5, it is possible to note that the cells treated with **BH** and **Basp** showed the same value in both cell proliferation and cell membrane integrity, while in the case of the cells treated with **AH,** the cytotoxic effects are lower and the cells are still capable of proliferating, like a consequence of the slower swelling behavior of **AH** hydrogel with respect to samples **BH** and **Basp**.

In fact, for the pea starch-based hydrogel, the release of LDH remained lower with respect to those of the two potato starch hydrogels, while the **AH** hydrogel allowed a certain proliferation after 24 h, albeit at very low values compared to the control.

The data at 48 h of incubation further confirmed what was previously observed. The values of LDH release by cells incubated with the hydrogels remain lower with respect to the control, while the cell proliferation goes to zero for all the hydrogels, as if the cells were in a sort of “frozen proliferative state” induced by the swallowing of the hydrogels that surrounds the cells and inhibits any sort of action. The phenomenon of the arrested cell cycle is known in oncology, and it can be triggered by chemical or genetic manipulations [67]. It has been recently demonstrated that various stressors can induce exit from the proliferative cell cycle into states of cell cycle arrest. An example is hypomitogenic stress, which can be induced by a lack of nutrients, inducing this arrest of the cell cycle, and successively, when cells regain their nutritional status, they can resume their replication [68]. By observing the effects induced by the hydrogels in cell culture, the data analysis allowed us to conclude that the materials have effectively hindered the availability of nutrients from the culture medium to the cells, thus arresting the cell cycle. These results were very interesting and similar to those conducted by applying Osmanthus-loaded PVP/PVA hydrogels, as it seems the treatment with such drug-loaded biomaterials indicated the block of the human keratinocyte (CAL-27) cell proliferation that has probably a physical mechanism rather than biological [69].

#### 2.3.2. Flow Cytometry Studies on Cell Apoptosis

Cellular apoptosis analyses were carried out using the flow cytometry technique, which allows the evaluation effect of newly synthesized hydrogels on the cellular response in subtler ways, identifying the percentage of viable cells, that are in early, late apoptosis, or even necrosis. Flow cytometry, as a part of the experimental design, was conducted to analyze the percentage of cells undergoing several phases of apoptosis and overcome the possibility that, even if cells are incorporated in the hydrogels, the cellular viability tests can give nonhomogenous results due to the dilutions of the hydrogels in cell culture media [70].

**Figure 6:**
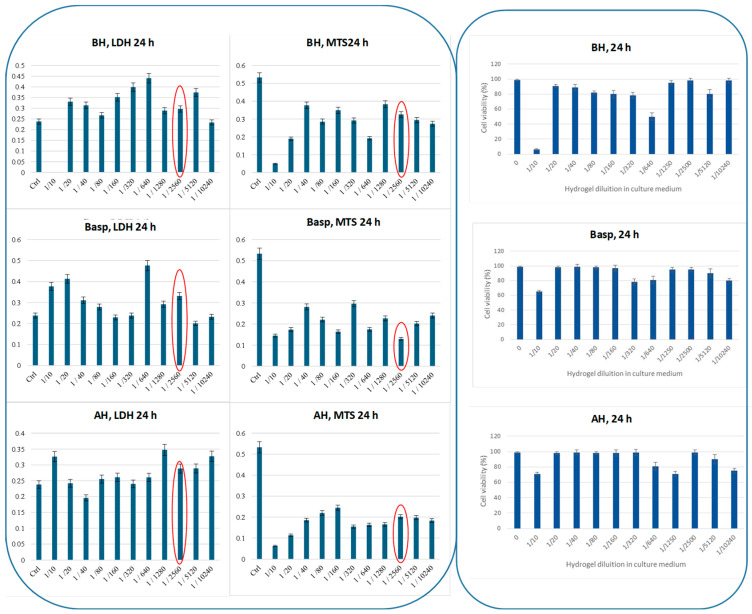
LDH, MTS (panel on the **left**), and viability assays (panel on the **right**) were registered at 24 h at different dilution ratios of hydrogel/medium (DMEM), on CAL-27 cell line. Conditions: CAL-27 cells, 5000 cells/well, in DMEM complete medium, 24 h of incubation under humidified atmosphere of air/CO_2_ (95:5) at 37 °C.

In order to identify the best dose-dependent condition that can favor apoptosis, a series of experiments were acquired varying the dilution ratio hydrogel:medium. Practically, hydrogels **AH**, **BH**, and **Basp** were incubated inside a 96-multiwell plate with DMEM cell medium through successive dilutions, harvesting 5000 cells per well. In each well, LDH and MTS assays (Figure 6) were evaluated at 24 h of incubation at 37 °C in a 5% CO_2_ atmosphere through a multiplate reader.

The results in Figure 6 highlighted that, at increasing dilutions, the cellular response did not appear to have a regular trend. In comparison to the data obtained for control cells (untreated), the best experimental conditions were chosen within the higher dilution value corresponding to the lower cytotoxicity and better proliferation (results in Figure 6 circled in red), and the more appropriated dilution ratio of hydrogel:medium was found to be 1:2560, for a concentration of 5000 cells per well, corresponding to 1 × 10^6^ cells per test tube for flow cytometry.

The calculated IC50 value (in Table S1), which is a parameter indicating the efficacy of a drug, has revealed that at 24 h all tested hydrogels had rather similar value.

Once the best experimental conditions were identified, the flow cytometry analysis was conducted, obtaining the quantification of four different cell populations through the comparison of the fluorescence intensities of the different markers used as protocol, such as viable cells (double negative), early apoptotic cells (positive for FITC-Annexin V and negative for PI), late apoptotic cells (positive for both PI and FITC-Annexin V), and necrotic cells.

Cells were visualized using an Annexin V FITC/PI(PE) dot-plot, and the distribution was evaluated by the intensity of Annexin V and PI labeling and successively plotted in two histograms: Annexin V FITC/Count and PI(PE)/Count. In the four quadrants of the Annexin V FITC/PI (PE) dot-plot are displayed the four distinct cell populations: viable cells, early apoptotic cells (EA), late apoptotic cells (LA), and necrotic cells (N). This subdivision in four different populations was possible because of the morphologic features of the apoptosis process, including loss of plasma membrane integrity (phospholipid phosphatidylserine translocation to the outer surface), one of the earliest events, condensation of the cytoplasm and nucleus, and internucleosomal cleavage of DNA.

Thus, cells that were considered viable were FITC-Annexin V and PI negative; cells that were in early apoptosis were FITC-Annexin V positive and PI negative, as mentioned above; and cells that were in late apoptosis or already dead were both FITC-Annexin V and PI positive.

**Figure 7:**
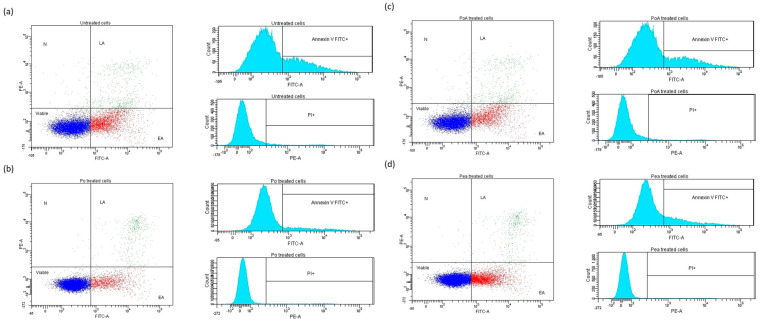
Representative dot-plots and histograms for untreated (**a**) and hydrogel-treated cell suspensions, ((**b**) = **BH**; (**c**) = **Basp**; (**d**) = **AH**). Dark blue—viable cells; red—early apoptotic cells; green—early apoptotic cells; light blue—cells.

In Figure 7, the flow cytometry dot-plots and histograms were illustrated; the quadrant, such as the marker for positivity, was compared to the four controls: negative, positive control for Annexin V, positive control for PI, and positive control for both markers. The controls were created for each type of cell suspension (treated and untreated cells).

Untreated cell controls stained with the markers showed the presence of two main populations of cells: viable cells not undergoing apoptosis (FITC-Annexin V and PI negative) and cells undergoing early apoptosis (FITC-Annexin V positive and PI negative), and in particular the latter in greater numbers than the others. A smaller population of cells positive for both markers, indicating the terminal phase of apoptosis (late apoptosis) or death (necrosis), was also observable (Figure 7a).

In the case of the cells treated with the hydrogels, **BH**, **Basp**, and **AH** (Figure 7b–d) the cell populations were very similar to the controls (Figure 7a). It was interesting to note that the hydrogels seemed to induce a lower “Early Apoptosis” and a higher “Late Apoptosis” in cells with respect to the untreated control.

The flow cytometry results, as the percentage of untreated and treated CAL-27 cell populations, are summarized in Table 1.

### 2.4. Quantitative NMR Studies on Starch-Based Hydrogels

**Figure 8:**
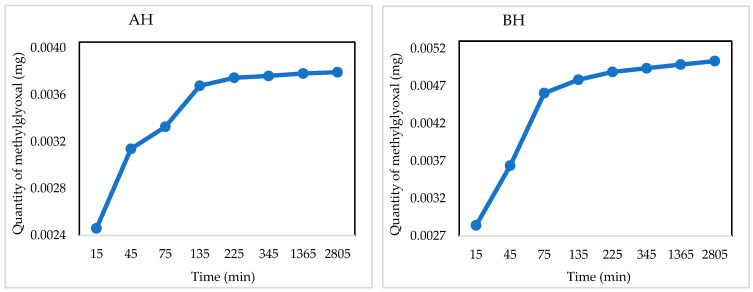
Quantitative release profile of methylglyoxal over time determined by NMR spectroscopy of loaded **AH** and **BH** hydrogels, respectively.

Quantitative NMR spectroscopy is a powerful analytical tool useful for evaluating the concentration of small molecules in solution. In this study, it was applied to evaluate the attitude of synthesized hydrogels to adsorb and release some drugs of biomedical interest over time. Hydrogels **AH** and **BH** were loaded with an aqueous solution of methylglyoxal, chosen as a model molecule for the functional groups able to interact with those on the hydrogels and even for the known antibacterial, antifungal, anti-inflammatory properties, and anticancer effects beyond being implicated in a variety of disorders [71,72]. The release profiles in Figure 8 were determined by acquiring a series of ^1^H experiments over time, as reported in Section 4.6.1. Results indicated a faster release profile for both the hydrogels in the first two hours, due to an easier release of methylglyoxal from the material surface, while successively a slower and prolonged trend was observed up to 46 h, as more time is required for the release of the drug from the inner part of the polymeric network, accordingly with literature data [63].

## 3. Conclusions

Starch-based hydrogels, as lightweight materials showing high porosity and drug release ability, were presented in this study as the first stage of development of a novel single-component formulation usable to encapsulate and deliver therapeutics. These hydrogels, derived from a sustainable reaction process, were tested for their effects on oral squamous cell carcinoma. The cytotoxicity of the hydrogels, assessed through LDH and MTS assays on CAL27 cell lines, revealed that the materials could inhibit cell proliferation without damaging the cell membrane, apparently inducing a “frozen proliferative state” due to the hydrogels’ swellability in the culture medium. Flow cytometry analysis showed reduced cell membrane damage in the initial contact with the hydrogels, followed by a progression to apoptosis in later stages. The manufactured hydrogels combined the dual ability to inhibit the proliferation and migration of CAL-27 cells and to deliver active drugs, as proven by NMR spectroscopy over time, and therefore could be recommended for therapeutic applications. Future research will aim to tailor the hydrogel design to deliver active molecules to the target disease in the optimal dose and throughout the entire treatment period, thus providing innovative solutions for personalized treatment.

## 4. Materials and Methods

### 4.1. Materials

Starch powders from pea pods (*Pisum sativum*) and potato peels (*Solanum tuberosum* L.) were provided by Emsland Group and used after purification.

Sodium chloride, sodium (meta)-periodate, ethylene glycol, L-Asparagine, and methylglyoxal were purchased from Merck (Merck Life Science, Via Monte Rosa, 93, 20149 Milano, Italy). Deuterated water, and TSP (trimethyl silyl propanoic acid) were purchased from CortecNet (7 Avenue du Hoggar, 91940 Les Ulis, France), and used as received. Dialysis membrane (MWCO = 7000 Da) were from BioSigma (Venice, Italy). CAL-27 cell cultures (oral squamous cell carcinoma) were purchased from American Type Culture Collection (10801 University Boulevard, Manassas, VA, USA) (CRL-2095™) and expanded according to the providers at the Immunology Department of the “Victor Babes” National Institute of Pathology in Bucharest, Romania. Bürker-Türk Chamber for the cell counting, and Leica inverted optical microscope for the monitoring were used. The CytoTox 96^®^ Non-Radioactive Cytotoxicity Assay kit (Promega; Madison, WI, USA) was used for the quantitative lactate dehydrogenase (LDH) measurement. The CellTiter 96^®^ AQueous One Solution Cell Proliferation Assay kit (Promega) was used for cell viability assays (MTS assay). Varioskan Thermo Fisher with multiwell plate, was used to evaluate and perform standard cell viability assays. BD Pharmingen™ FITC Annexin V Apoptosis Detection Kit-I, (Franklin Lakes, NJ, USA) was used for cell apoptosis studies in Flow Cytometry, (BD Becton Dickinson FACSCanto II flow cytometer). Data were processed thought the BD FACSDiva v.6.1 software. TEM analyses used solution of 4% glutaraldehyde (Agar scientific, London, UK) and 0.1% Ruthenium Red (Ladd Research Industries, Burlington, VT, USA) in pure water, solution of 1% osmium tetroxide (Agar scientific, London, UK), 0.15% Ruthenium Red and 0.08 M cacodylate (Agar scientific, London, UK), ethanol, propylene oxide, epoxy resin (Epon 812) (Agar scientific, London, UK). Ultramicrotome Leica EM UC7 (Leica Microsystems GmbH, Wetzlar, Germany) was used for thin sections, and solutions of uranyl acetate and lead citrate (Electron Microscopy Sciences, Hatfield, PA, USA) for staining. Transmission Electron Microscope (Morgagni268, FEI, Eindhoven, The Netherlands) at 80 kV and MegaView III CCD using iTEM SIS, software (Olympus Soft Imaging Software, Munster, Germany) were used for visualization.

### 4.2. Synthesis of Starch Hydrogels

Hydrogels from pea and potato starch, namely **AH**, and **BH**, were synthesized following the methodology reported in a previous study [63]. Briefly, starch, after purification, was suspended in water at 45 °C (2.0 g in 70 mL of Milli-Q water) under constant stirring, and then sodium (meta)-periodate was added (0.9 g, ca 0.35 molar ratio based on starch), and the reaction was carried out in these experimental conditions for 5 h. The reactor was covered with an aluminum foil to prevent the photo-induced decomposition of periodate ions. The reaction was interrupted by adding ethylene glycol (2.0 mL) to the mixture, which was successively kept at room temperature, and stirred overnight. Finally, the mixture was first kept at −18 °C for 24 h, and then the solid was thawed and washed several times with MilliQ water, or alternatively dialyzed against deionized water for 2 days using a cellulose membrane, to eliminate residual NaIO_4_ and ethylene glycol. The recovered hydrogels were stored at −18 °C. The materials were then spectroscopically characterized by NMR and FTIR.

### 4.3. Synthesis of Asparagine-Crosslinked Hydrogels

To the oxidized potato starch slurry (1 g) described in Section 4.2, 1 mL of a water suspension of asparagine (40 mg/mL) was added, and the mixture was stirred 2 h at room temperature, then kept overnight at the same temperature for allowing the complete Schiff-base reaction among the dialdehyde groups, of the oxidized starch, and the amine group of asparagine. The reaction mixture was kept at −18 °C for 24 h and, after thawing, it was washed following the same protocol illustrated in Section 4.2. The new formed imine group was observed through NMR and by FTIR spectroscopy. Once the occurrence of the Schiff-base reaction was determined, the cross-linked hydrogels, namely **Basp**, were kept at −18 °C for 24 h, and the resulting hydrogels were thawed and washed several times with MilliQ water.

### 4.4. Swelling Degree (SD)

Hydrogels were cored into cylindrical samples with a final diameter of 10 mm that were weighted, gently dried on filter paper to remove the water, and reweighted to determine the swelling degree according to Equation (1):

SD (%) = 100 × (Ww − Wd)/Wd(1)

Ww and Wd are, respectively, the weights of wet and dry samples. Tests were carried out in triplicate.

The water retention capacity, meaning the swelling degree evaluated at regular intervals of time, was calculated applying Equation (1) by periodically weighting the samples after the contact with water and until the weight of wet and dry samples remained constant.

### 4.5. FTIR-Fourier Transform Infrared Spectroscopy

The molecular structure of all materials, as native, oxidized, and cross-linked starch, was investigated though FTIR analysis. Measurements were acquired on KBr disc, by mixing 1 mg of dried sample with the right amount of KBr. The spectra were recorded with a scanning wavelength range of 4000–500 cm^−1^.

### 4.6. NMR-Solution Nuclear Magnetic Resonance

NMR experiments were recorded on a Bruker spectrometer operating at 11.7 T, 500 MHz, and equipped with a 5 mm probe and gradient unit on z. Samples were prepared by dissolving 5.0 mg of materials into 750 µL of D_2_O at room temperature, and adding 10 µL of a TSP/water solution (TSP 3-(Trimethylsilyl)propionic acid, sodium salt 0.05 wt%), as an internal standard.

#### 4.6.1. qNMR-Quantitative Nuclear Magnetic Resonance

The attitude of prepared hydrogels to adsorb and deliver drugs over time was determined by quantitative NMR spectroscopy. A water solution of methylglyoxal was chosen as the model molecule, for conducting these studies, as shown in SI section. The direct quantification of methylglyoxal by means of qNMR was possible because, in the proton spectrum, the area of the signals is directly proportional to the number of protons present in the active volume of the sample. Methylglyoxal evaluation was performed by comparing the signal of the internal standard (TSP at δ = 0.00 ppm), with those of the methyl protons of both mono- and di-hydrated methylglyoxal forms (at δ = 2.30 ppm and 1.38 ppm, respectively) [73,74], by applying Equation (2):

[mM]_m_ = I_m_/H_m_ [mM]_st_ × H_st_/I_st_ (2)

where [mM]_m_ and [mM]_st_ are the millimolar concentrations of methylglyoxal and the standard solution of TSP; I_m_, and I_st_ are the integral values of the signals assigned to the methylglyoxal in the mono- and dihydrated forms and the TSP, while H_m_ and H_st_ are the number of the protons generating the signals [75]. The ^1^H spectrum of methylglyoxal is shown in Figure S1.

### 4.7. TEM—Transmission Electron Microscopy

To determine the hydrogels’ microstructures, they were lyophilized, and the TEM analysis was carried out on the respective cryogels. The materials were prefixed in a solution of 4% glutaraldehyde and 0.1% Ruthenium Red in pure water, then washed in pure water and postfixed in a solution of 1% osmium tetroxide, 0.15% Ruthenium Red and 0.08 M cacodylate. Dehydration was performed in several concentrations of ethanol and Propylene Oxide, followed by embedding in the epoxy resin. Thin sections of 80 nm thickness were cut at room temperature on a Leica EM UC7 ultramicrotome. The double staining of thin sections on grids was performed with solutions of uranyl acetate and lead citrate. The sections were visualized under a Transmission Electron Microscope (Morgagni268, FEI, Eindhoven, The Netherlands) and MegaView III CCD using iTEM SIS software (Olympus Soft Imaging Software, Munster, Germany) was used for data acquisition.

### 4.8. Cellular Viability Tests

Cell viability assays are mandatory tests used to measure the cells response to extracellular stimuli, chemical agents, or therapeutic treatments by monitoring the cytotoxic effects. Herein, cellular viability was evaluated in terms of the cell’s response after the contact with the synthesized hydrogels.

All the materials, after a proper sterilization procedure applying UV radiation for 30 min, were tested on CAL-27 cell cultures, which represent an excellent model for screening the cell viability and migration because this cell line is characterized by an active proliferation rate [76].

#### 4.8.1. Cell Culture Preparation

CAL-27 cell cultures were maintained as instructed by the producer, and then the cells rough viability was evaluated in Bürker-Türk Chamber, staining was performed with the vital dye methylene blue and automatically counted in Countess 3 Automated Cell Counter (ThermoFischer, 24 Preciziei Blvd, Bucharest, 062204 Romania). Their growth monitoring and expansion was daily checked in an inverted optical microscope. CAL-27 cells were cultivated in DMEM (Dulbecco’s Modified Eagle Medium) complete medium in tissue-culture-treated Corning^®^ flasks (Sigma-Aldrich, St. Louis, MO, USA), under a humidified atmosphere of air/CO_2_ (95:5) at 37 °C in an incubator.

#### 4.8.2. LDH Assay

Lactate dehydrogenase (LDH) activity assay evaluates the cellular membrane integrity. The test actually evaluates the concentration of LDH in cell culture supernatants when cells are subjected to various treatments. The principle of the test resides on LDH, which is a cytosolic oxidoreductase enzyme catalyzing the interconversion of pyruvate to lactate, being present in all cells. When the cell membrane is damaged due to the infliction of the treatment, LDH spills into the supernatant and can be detected in the cell culture medium.

Upon contact with the newly synthetized hydrogels, we have tested the LDH release at 24 and 48 h of incubation with **AH**, **BH**, and **Basp** starch hydrogels.

The method used was the CytoTox 96^®^ Non-Radioactive Cytotoxicity Assay, a colorimetric alternative to ^51^Cr release cytotoxicity assays. The CytoTox 96^®^ Assay quantitatively measures LDH released upon cell lysis, in much the same way as ^51^Cr is released in radioactive assays. The half-life of LDH that has been released from cells into the surrounding medium is approximately 9 h. Released LDH in culture supernatants is measured with a 30-min coupled enzymatic assay, which results in the conversion of a tetrazolium salt (iodonitrotetrazolium violet; INT) into a red formazan product. The amount of color formed is proportional to the number of lysed cells. The absorbance signal is measured at 490 nm in a 96-well plate reader.

#### 4.8.3. MTS Assay

Cellular metabolic activity is a direct indicator of cell viability (MTS assay) and an indirect measure of proliferation assessing the cytotoxicity of various compounds.

MTS assay was evaluated after 24 and 48 h of cellular treatment with the synthesized hydrogels, by using the CellTiter 96^®^ AQueous One Solution Cell Proliferation Assay (Promega). This is also a colorimetric method for evaluating the NADPH or NADH produced by dehydrogenase enzymes in metabolically active cells. Assays are performed by adding 40 µL of CellTiter 96^®^ AQueous One Solution Reagent directly to culture wells, incubating for 1–4 h and then recording the absorbance at 490 nm with a 96-well plate reader. The quantity of formazan product is directly proportional to the number of living cells in culture, hence to their metabolic activity.

#### 4.8.4. Cell Apoptosis Studies

Experiments on cell viability and apoptosis were carried out using Flow Cytometry assessing of the effect of AH, BH, and AspH starch hydrogels on CAL-27.

Cell viability analysis was performed using FITC Annexin V Apoptosis Detection Kit I, and BD FACSCanto II flow cytometer. Briefly, the assay evaluates cells undergoing the stages of apoptosis. In the early stages of apoptosis, phosphatidylserine residues are exposed on the external side of the membrane lipid bilayer and can bind to FITC-conjugated Annexin V introduced into the system, thus highlighting the early stages of apoptosis (cells are negative for PI labeling). With increased plasma membrane permeability (advanced apoptosis), PI binds intracellularly to cell DNA, identifying late apoptotic cells, positive for both PI and FITC- Annexin V. In the necrotic stage, cells are positive only for PI while the cellular membrane is damaged without expressing phosphatidylserine residues.

Cells (untreated and treated with our biomaterials for 24 h) were washed twice with PBS and suspended in Annexin V Binding Buffer 1×, and then distributed in 4 tubes (100 µL/tube, final concentration 1 × 10^6^ cells/mL) and labeled as: negative control (unstained cells); positive control for Annexin V FITC; positive control for PI; sample (cells stained with Annexin V FITC and PI).

Additionally, 5 µL Annexin V FITC, and 5 µL of PI, were poured into their respective tubes, gently shaken, and incubated for 15 min at room temperature in the dark, and then 400 µL of Annexin V Binding Buffer 1X was added to each tube and analyzed on the flow cytometer within one hour.

Cell cultures were performed according to the producer, and all the standard safety measures that are implemented in Victor Babes National Institute of Pathology in line with ISO 9001:2015 were followed [77].

#### 4.8.5. Statistics

For statistics we have used the XLMiner Analysis Tool pack within Microsoft Excell software. For IC50 calculations, we have fitted for our data an x-y plot using cellular viabilities and fit the data with a linear regression. IC50 value was calculated with the formula IC50 = (0.5 − b)/a.

## Figures and Tables

**Table 1:** Percentage of untreated and treated CAL-27 cell population obtained through flow cytometry data analysis.

Cells/Treatments	Viable	Early Apoptosis	Late Apoptosis	Necrosis
Control	72.2	23.3	4.0	0.6
**AH** treated	79.9	17.8	2.2	0.1
**BH** treated	86.6	10.6	2.6	0.1
**Basp** treated	75.9	19.4	4.2	0.6

## Data Availability

Data are contained within the article and Supplementary Materials.

## Supplementary Materials

The following supporting information can be downloaded at: https://www.mdpi.com/article/10.3390/molecules29225463/s1, Table S1. Statistical parameters for the LDH and MTS assays at 24 h of incubation; qNMR: quantitative NMR details; Figure S1. ^1^H NMR spectrum of methylglyoxal at 600 MHz in D_2_O; Figure S2. FTIR spectra with an expaded region; Figure S3. Macroscopical pictures of the produced materials. Viscosity measurements.
