# Supplementary material for: Single-Component Starch-Based Hydrogels for Therapeutic Delivery
Source: Molecules. 2024 Nov 20;29(22):5463. doi: 10.3390/molecules29225463 (PMC11597573; doi:10.3390/molecules29225463)
Supplement: Supplementary file 1 [file molecules-29-05463-s001.zip › molecules-3244379-supplementary.pdf]

Single-component Starch-based hydrogels for therapeutic delivery

Alfio Pulvirenti [1], Antonella Caterina Boccia [1*], Carolina Constantin [2,3], Mihaela Surcel [2], Adriana Munteanu [2], Victor-Eduard Peteu [2,4] and Monica Neagu [2]

[1] Istituto di Scienze e Tecnologie Chimiche (SCITEC) "Giulio Natta", C.N.R., Via Alfonso Corti 12, 20133 Milano, Italy; alfio.pulvirenti@scitec.cnr.it
[2] Victor Babes" National Institute of Pathology, 99-101 Splaiul Independenței, Bucharest, Romania caroconstantin@gmail.com ; msurcel2002@yahoo.com ; iadinuta@yahoo.com ; neagu.monica@gmail.com
[3] Colentina Clinical Hospital, 19-21, Sos Stefan Cel Mare, Bucharest, Romania
[4] Politechnica University of Bucharest, 313 Splaiul Independenței, Bucharest, Romania. peteuvictoreduard@gmail.com
* Istituto di Scienze e Tecnologie Chimiche (SCITEC) "Giulio Natta", C.N.R., Via Alfonso Corti 12, 20133 Milano, Italy; antonella.boccia@scitec.cnr.it

Table S1. Statistical parameters for the LDH and MTS assays at 24h of incubation

Parameter	BH LDH 24h	BH MTS 24 h	Basp LDH 24h	Basp MTS 24h	AH LDH 24h	AH MTS 24h
Mean	0.299818	0.273909	0.307182	0.205	0.27562	0.171733
Standard Error	0.03498	0.029487	0.025938	0.01618	0.013585	0.015157
Standard Deviation	0.116014	0.097797	0.086025	0.053664	0.045056	0.050271
Sample Variance	0.013459	0.009564	0.0074	0.00288	0.00203	0.002527
Confidence Level (95%)	0.07794	0.065701	0.057793	0.036052	0.030269	0.033773

Calculation of the IC50 for our hydrogels upon the cellular viability at 24h has revealed the following values: for **BH** IC50=1/45.95, for **Basp** IC50 = 1/70.15, for **AH** IC50=1/78.36. It is interesting that all the tested hydrogels have rather similar IC50.

1.1 qNMR*: quantitative NMR details*

Quantitative acquisition parameters for ^{1}H spectra: 90° pulse as calculated for each sample; relaxation delay 40.0 s. Spectral width 8400 Hz; number of transient 128. Data processing: exponential line broadening of 0.1 Hz was applied as resolution enhancement function; zero-filling to 32 K prior FT.

1.2 Hydrogels as delivery systems: the methodology for collecting the NMR samples.

Hydrogels were carved to obtain plugs of 10 mm of diameters, and transferred into an Eppendorf tube containing 150 μL of methylglyoxal solution, at room temperature for 30 minutes. After this interval, the loaded plugs were gently passed on filter paper and then placed into 550 μL of fresh deuterated water at regular interval time (15, 30, 30, 60, 90, 120, 1020, and 1440 minutes as contact time), then the solutions were collected for data acquisition. Data were duplicated for both hydrogels.

Preparation of Methylglyoxal solution [1 mM]

The methylglyoxal solution was prepared by diluting 2.0 mL of pure methylglyoxal in 2.0 mL of deuterated water to obtain a solution 8.17 M that was successively diluted in D_2O to obtain 2.0 mL of 1 mM methylglyoxal solution.

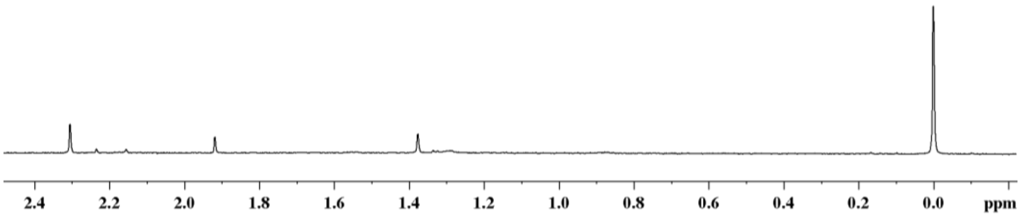

Figure S1 ^1H NMR spectrum of methylglyoxal at 600MHz in D_2O.

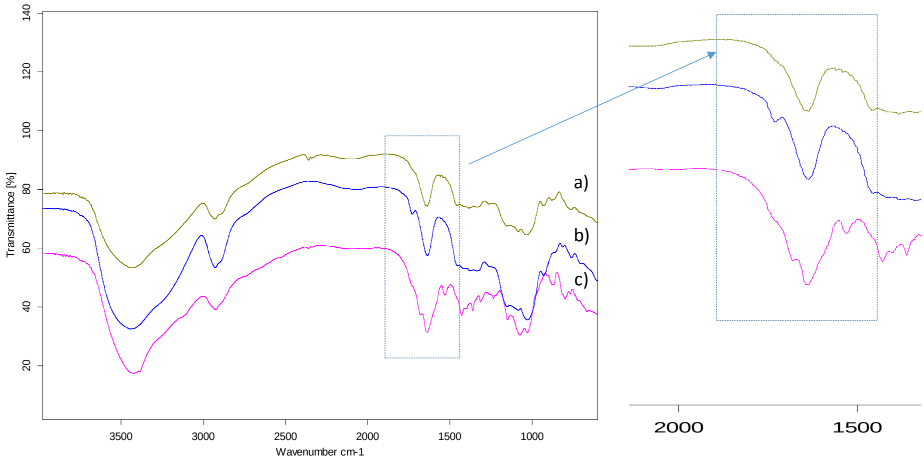

Figure S2. FTIR spectra of: a) native pea starch; b) dialdehyde starch; c) crosslinked starch with asparagine. In the expanded regior are highlighted the stretching vibrations of C=O and C=N, as new formed groups.

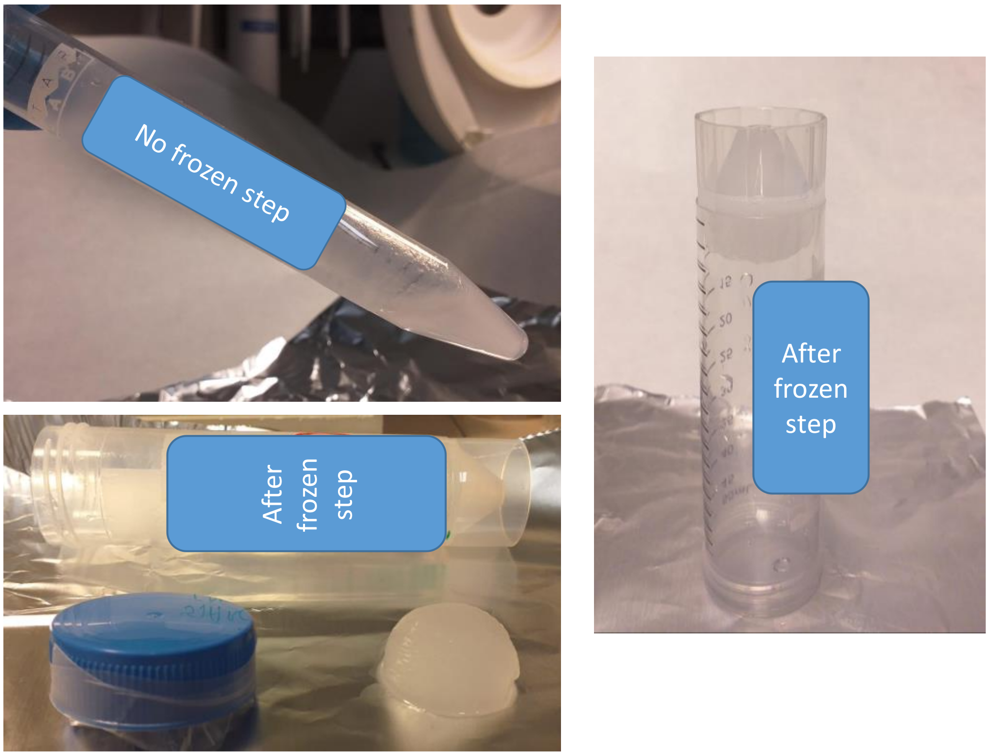

Figure S3. Macroscopical pictures of the produced materials depending on whether or not a frozen step was applied.
